# Parental atopy and risk of atopic dermatitis in the first two years of life in the BASELINE birth cohort study

**DOI:** 10.1111/pde.15090

**Published:** 2022-07-25

**Authors:** Cathal O'Connor, Vicki Livingstone, Jonathan O'. B. Hourihane, Alan D. Irvine, Geraldine Boylan, Deirdre Murray

**Affiliations:** ^1^ Paediatrics and Child Health Cork University Hospital Cork Ireland; ^2^ INFANT Research Centre University College Cork Cork Ireland; ^3^ Paediatrics and Child Health Royal College of Physicians of Ireland Dublin Ireland; ^4^ Dermatology Children's Health Ireland at Crumlin Dublin Ireland; ^5^ Clinical Medicine Trinity College Dublin Ireland

**Keywords:** atopic dermatitis, atopy, family history, genetics

## Abstract

**Background:**

Atopic dermatitis (AD) has a strong genetic basis. The objective of this study was to assess the association between parental atopy and AD development by 2 years.

**Methods:**

A secondary data analysis of the BASELINE Birth Cohort study was performed (*n* = 2183). Parental atopy was self‐reported at 2 months. Infants were examined for AD by trained health care professionals at 6, 12, and 24 months. Variables extracted from the database related to skin barrier function, early skincare, parental atopy, and AD. Statistical analysis adjusted for potential confounding variables.

**Results:**

Complete data on AD status were available for 1505 children at 6, 12, and 24 months. Prevalence of AD was 18.6% at 6 months, 15.2% at 12 months, and 16.5% at 24 months. Adjusted odds ratios (95% CIs) following multivariable analysis were 1.57 (1.09–2.25) at 6 months and 1.66 (1.12–2.46) at 12 months for maternal AD; 1.90 (1.28–2.83) at 6 months and 1.85 (1.20–2.85) at 24 months for paternal AD; 1.76 (1.21–2.56) at 6 months and 1.75 (1.16–2.63) at 12 months for maternal asthma; and 1.70 (1.19–2.45) at 6 months, 1.86 (1.26–2.76) at 12 months, and 1.99 (1.34–2.97) at 24 months for paternal asthma. Parental rhinitis was only associated with AD with maternal rhinitis at 24 months (aOR (95% CI): 1.79 (1.15–2.80)).

**Conclusion:**

Parental AD and asthma were associated with increased risk of objectively diagnosed AD in offspring in this contemporary cohort.

## 1 INTRODUCTION

Atopic dermatitis (AD) affects one in five children,^1^ usually starts in the first year of life, and commonly persists into adulthood.^2^ The pathophysiology of AD is complex, involving skin barrier dysfunction,^3^ aberrant immune responses,^4^ and environmental factors such as microbial exposure.^5^ Loss of function mutations in *FLG*, the gene encoding filaggrin, represent the greatest genetic risk factor for development of AD.^6^

Previous studies have shown differing odds ratios for AD in offspring of parents with atopic disease, although parental AD has been consistently associated with increased risk.^7^, ^8^, ^9^, ^10^ The Avon longitudinal study of parents and children (ALSPC) showed a strong association between parental AD and childhood AD, with an odds ratio of 1.69 (95% confidence interval 1.47–1.95) for maternal AD, 1.74 (1.44–2.09) for paternal AD, and 2.72 (2.09–3.53) for biparental AD.^7^ The PARIS prospective birth cohort study showed that parental atopy (AD and/or asthma and/or rhinitis) was associated with an odds ratio of 2.31 (1.28–4.16) for severe AD.^11^ The Protection Against Allergy Study in Rural Environments (PASTURE) birth cohort study showed that having one parent with atopy (AD and/or asthma and/or rhinitis) was associated with an odds ratio of 1.36 (0.84–2.20) for early transient AD, 2.15 (1.15–4.03) for early persistent AD, and 1.58 (0.83–3.03) for late AD; while having two parents with atopy was associated with an odds ratio of 2.46 (1.27–4.76) for early transient AD, 5.35 (2.52–11.36) for early persistent AD, and 2.41 (0.95–6.09) for late AD.^12^ Some studies have suggested that maternal atopy is more strongly associated with AD,^10^, ^13^ while others have not identified a difference in risk between maternal or paternal atopy.^7^, ^8^

We aimed to assess the impact of maternal and paternal atopic disease on AD outcomes in offspring in early life in a large observational birth cohort study.

## 2 METHODS

### 2.1 Study subjects

This study was a secondary analysis of the Cork Babies After Scope: Evaluating the Longitudinal Impact Using Neurological and Nutritional Endpoints (BASELINE) Birth Cohort study.^14^ The purpose of the BASELINE study was to examine the effects of environmental factors during pregnancy and infancy on childhood health and development. The BASELINE study recruited healthy first‐born term babies in Cork, Ireland, from August 2009 through to October 2011. Ethical approval was granted by the Clinical Research and Ethics Committee of the Cork Teaching Hospitals [ref ECM5(9) 01/07/2008]. Skin barrier assessment was performed on 2183 infants at birth and throughout early life by measuring transepidermal water loss (TEWL) using a validated open chamber system (Tewameter TM 300; Courage + Khazaka Electronic, Cologne, Germany).

### 2.2 Atopy and atopic dermatitis assessments

All infants had assessments at birth, 2 months, 6 months, 12 months, and 24 months involving parental questionnaires and physical assessment. Self‐reported parental atopy (AD, asthma, or allergic rhinitis) was investigated by the questions

- DO or DID you ever suffer from eczema (atopic dermatitis)?
- DO or DID you ever suffer from asthma?
- DO or DID you ever suffer from pollen‐related rhinitis (“hayfever”)?

Parental atopy was self‐reported at 2 months. Parents were asked at 2 months if the infant had an “itchy rash on the face or in the folds of the arms or legs,” as a screening question for AD. Experienced health care personnel diagnosed AD at 6, 12, and 24 months according to the UK Working Party diagnostic criteria.^15^

### 2.3 Statistical analysis

Categorical variables were described using frequency (percentage). Univariable and multivariable logistic regression models were used to investigate relationships between maternal and paternal atopic conditions, potential confounding variables and the presence of AD at 6 months, 12 months, and 24 months of age separately, and at all‐time points. The potential confounders included were sex, birth weight, transepidermal water loss (TEWL) at 2 months, parent‐reported “itchy rash” at 2 months, emollient bathing at 2 months, frequency of bathing at 2 months, and frequency of emollient application at 2 months. For all independent variables, the unadjusted and adjusted odds ratios (ORs) and 95% confidence intervals (95% CIs) are presented.

Prior to performing the multivariable logistic regression analyses, multicollinearity among the independent variables was tested using the variance inflation factor (VIF). All tests were two‐sided and a *p*‐value <.05 was considered statistically significant. Statistical analysis was performed using Stata (version 15.1, StataCorp LP, College Station, TX).

## 3 RESULTS

**FIGURE 1:**
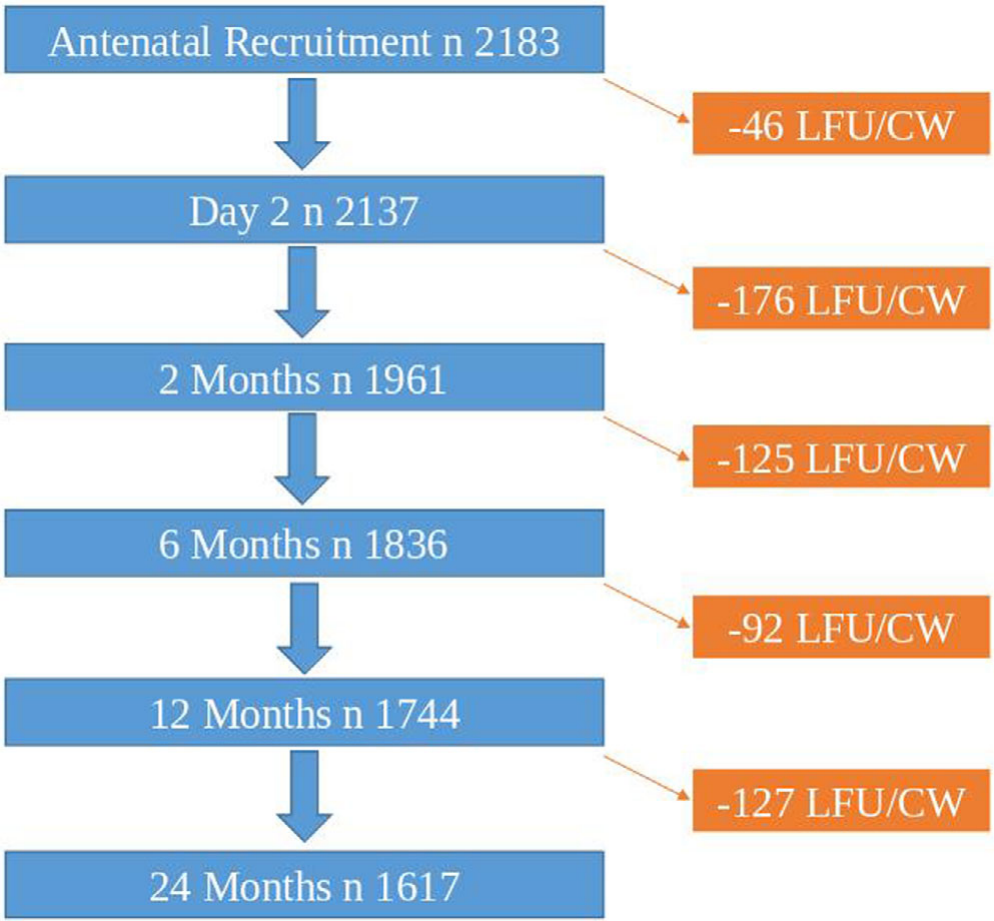
Flow diagram showing each stage of the BASELINE study. *n*, number of infants; LFU, lost to follow up; CW, consent withdrawn

A flow diagram outlining study recruitment is shown in Figure 1. Complete data on AD status were available for 1505 children at 6, 12, and 24 months. Prevalence of AD was highest at 6 months (18.6%), decreasing to 15.2% at 12 months, and increasing to 16.5% at 24 months.

### 3.1 Relationship between parental atopy and AD in first 2 years of life

Parental atopic disease (either maternal or paternal or biparental history of AD and/or asthma and/or rhinitis) was associated with AD in both univariable and multivariable analysis (*n* = 1296). Based on the multivariable analysis, the odds of AD in the first 2 years of life were higher among infants whose mother or father or both parents had a history of atopy {adjusted OR (95% CI): 2.26 (1.75–2.91), *p* < .001}. Maternal atopic disease (AD and/or asthma and/or rhinitis) was also associated with AD in both univariable and multivariable analysis. Based on the multivariable analysis, the odds of AD in the first 2 years of life were higher among infants whose mothers had a history of atopy {adjusted OR (95% CI): 1.70 (1.31–2.22), *p* < .001}. Paternal atopic disease (AD and/or asthma and/or rhinitis) was also associated with AD in both univariable and multivariable analysis. Based on the multivariable analysis, the odds of AD in the first 2 years of life were higher among infants whose fathers had a history of atopy {adjusted OR (95% CI): 1.99 (1.51–2.61), *p* < .001}.

### 3.2 Relationship between specific parental atopic disease and AD at 6 months

At 6 months, maternal AD and asthma and paternal AD and asthma were significantly associated with AD, in both univariable and multivariable analysis (Table 1). Based on the multivariable analysis, the odds of AD at 6 months were higher among infants whose mothers had AD {adjusted OR (95% CI): 1.57 (1.09–2.25)} and among infants whose mothers had asthma {adjusted OR (95% CI): 1.76 (1.21–2.56)}. The odds of AD at 6 months were also higher among infants whose fathers had AD {adjusted OR (95% CI): 1.90 (1.28–2.83)} and among infants whose fathers had asthma {adjusted OR (95% CI): 1.70 (1.19–2.45)}. Neither maternal nor paternal rhinitis were associated with increased risk of AD at 6 months.

**TABLE 1:** Results of the univariable and multivariable logistic regression analyses of family history with AD at 6 months as the dependent variable, *n* = 1538

	Eczema at 6 months		Univariable analysis		Multivariable analysis^a^	
	Yes (*n* = 276) *n* (%)	No (*n* = 1262) *n* (%)	OR (95% CI)	*p*‐value	OR (95% CI)	*p*‐value
*Maternal AD*						
No	217 (78.6)	1110 (88.0)	1	<.001	1	.014
Yes	59 (21.4)	152 (12.0)	**1.99** (1.42–2.77)		**1.57** (1.09–2.25)	
*Maternal asthma*						
No	216 (78.3)	1098 (87.0)	1	<.001	1	.003
Yes	60 (21.7)	164 (13.0)	**1.86** (1.34–2.59)		**1.76** (1.21–2.56)	
*Maternal rhinitis*						
No	237 (85.9)	1125 (89.1)	1	.123	1	.773
Yes	39 (14.1)	137 (10.9)	1.35 (0.92–1.98)		1.06 (0.70–1.63)	
*Paternal AD*						
No	226 (81.9)	1156 (91.6)	1	<.001	1	.001
Yes	50 (18.1)	106 (8.4)	**2.41** (1.67–3.48)		**1.90** (1.28–2.83)	
*Paternal asthma*						
No	214 (77.5)	1088 (86.2)	1	<.001	1	.004
Yes	62 (22.5)	174 (13.8)	**1.81** (1.31–2.51)		**1.70** (1.19–2.45)	
*Paternal rhinitis*						
No	252 (91.3)	1165 (92.3)	1	.573	1	.328
Yes	24 (8.7)	97 (7.7)	1.14 (0.72–1.82)		0.77 (0.46–1.30)	

*Note:* Bold values are those which were statistically significant (*p* value < 0.05).

^a^ Includes the variables listed and sex, birth weight, and TEWL, “itchy rash,” emollient bathing, frequency of bathing, and frequency of emollient application at 2 months.

### 3.3 Relationship between specific parental atopic disease and AD at 12 months

At 12 months, maternal AD and asthma and paternal AD and asthma were significantly associated with AD, in the univariable analysis (Table 2). Paternal AD was no longer significantly associated with AD following multivariable analysis (*p* = .060). Based on the multivariable analysis, the odds of AD at 12 months were higher among infants whose mothers had AD {adjusted OR (95% CI): 1.66 (1.12–2.46)} and among infants whose mothers had asthma {adjusted OR (95% CI): 1.75 (1.16–2.63)}. The odds of AD at 12 months were also higher among infants whose fathers had asthma {adjusted OR (95% CI): 1.86 (1.26–2.76)}. Neither maternal nor paternal rhinitis were associated with increased risk of AD at 12 months.

**TABLE 2:** Results of logistic regression analyses of family history with AD at 12 months as the dependent variable, *n* = 1452

	Eczema at 12 months		Univariable analysis		Multivariable analysis^a^	
	Yes (*n* = 219) *n* (%)	No (*n* = 1233) *n* (%)	OR (95% CI)	*p*‐value	OR (95% CI)	*p*‐value
*Maternal AD*						
No	172 (78.5)	1088 (88.2)	1	<.001	1	.011
Yes	47 (21.5)	145 (11.8)	**2.05** (1.42–2.96)		**1.66** (1.12–2.46)	
*Maternal asthma*						
No	172 (78.5)	1068 (86.6)	1	.002	1	.008
Yes	47 (21.5)	165 (13.4)	**1.77** (1.23–2.54)		**1.75** (1.16–2.63)	
*Maternal rhinitis*						
No	190 (86.8)	1094 (88.7)	1	.402	1	.831
Yes	29 (13.2)	139 (11.3)	1.20 (0.78–1.84)		0.95 (0.59–1.53)	
*Paternal AD*						
No	181 (82.6)	1118 (90.7)	1	<.001	1	.060
Yes	38 (17.4)	115 (9.3)	**2.04** (1.37–3.04)		1.52 (0.98–2.36)	
*Paternal asthma*						
No	168 (76.7)	1058 (85.8)	1	.001	1	.002
Yes	51 (23.3)	175 (14.2)	**1.84** (1.29–2.61)		**1.86** (1.26–2.76)	
*Paternal rhinitis*						
No	202 (92.2)	1134 (92.0)	1	.893	1	.131
Yes	17 (7.8)	99 (8.0)	0.96 (0.56–1.65)		0.63 (0.35–1.15)	

*Note:* Bold values are those which were statistically significant (*p* value < 0.05).

^a^ Includes the variables listed and sex, birth weight, and TEWL, “itchy rash,” emollient bathing, frequency of bathing, and frequency of emollient application at 2 months.

### 3.4 Relationship between specific parental atopic disease and AD at 24 months

At 24 months, maternal AD, asthma and rhinitis and paternal AD and asthma were significantly associated with AD, in the univariable analysis (Table 3). Maternal AD (*p* = .359) and asthma (*p* = .216) were no longer significantly associated with AD following multivariable analysis. Based on the multivariable analysis, the odds of AD at 24 months were higher among infants whose mothers had rhinitis {adjusted OR (95% CI): 1.79 (1.15–2.80)}. The odds of AD at 24 months were also higher among infants whose fathers had AD {adjusted OR (95% CI): 1.85 (1.20–2.85)} and among infants whose fathers had asthma {adjusted OR (95% CI): 1.99 (1.34–2.97)}. Paternal rhinitis was not associated with risk of AD at 24 months.

**TABLE 3:** Results of the univariable and multivariable logistic regression analyses of family history with AD at 24 months as the dependent variable, *n* = 1332

	Eczema at 24 months		Univariable analysis		Multivariable analysis^a^	
	Yes (*n* = 210) *n* (%)	No (*n* = 1122) *n* (%)	OR (95% CI)	*p*‐value	OR (95% CI)	*p*‐value
*Maternal AD*						
No	172 (81.9)	983 (87.6)	1	.026	1	.359
Yes	38 (18.1)	139 (12.4)	**1.56** (1.05–2.32)		1.22 (0.80–1.86)	
*Maternal asthma*						
No	169 (80.5)	972 (86.6)	1	.020	1	.216
Yes	41 (19.5)	150 (13.4)	**1.57** (1.07–2.30)		1.32 (0.85–2.03)	
*Maternal rhinitis*						
No	171 (81.4)	1006 (89.7)	1	.001	1	.010
Yes	39 (18.6)	116 (10.3)	**1.98** (1.33–2.94)		**1.79** (1.15–2.80)	
*Paternal AD*						
No	169 (80.5)	1022 (91.1)	1	<.001	1	.006
Yes	41 (19.5)	100 (8.9)	**2.48** (1.66–3.69)		**1.85** (1.20–2.85)	
*Paternal asthma*						
No	157 (74.8)	967 (86.2)	1	<.001	1	.001
Yes	53 (25.2)	155 (13.8)	**2.11** (1.48–3.00)		**1.99** (1.34–2.97)	
*Paternal rhinitis*						
No	188 (89.5)	1035 (92.2)	1	.188	1	.695
Yes	22 (10.5)	87 (7.8)	1.39 (0.85–2.28)		0.89 (0.51–1.56)	

*Note:* Bold values are those which were statistically significant (*p* value < 0.05).

^a^ Includes the variables listed and sex, birth weight, and TEWL, “itchy rash,” emollient bathing, frequency of bathing, and frequency of emollient application at 2 months.

**FIGURE 2:**
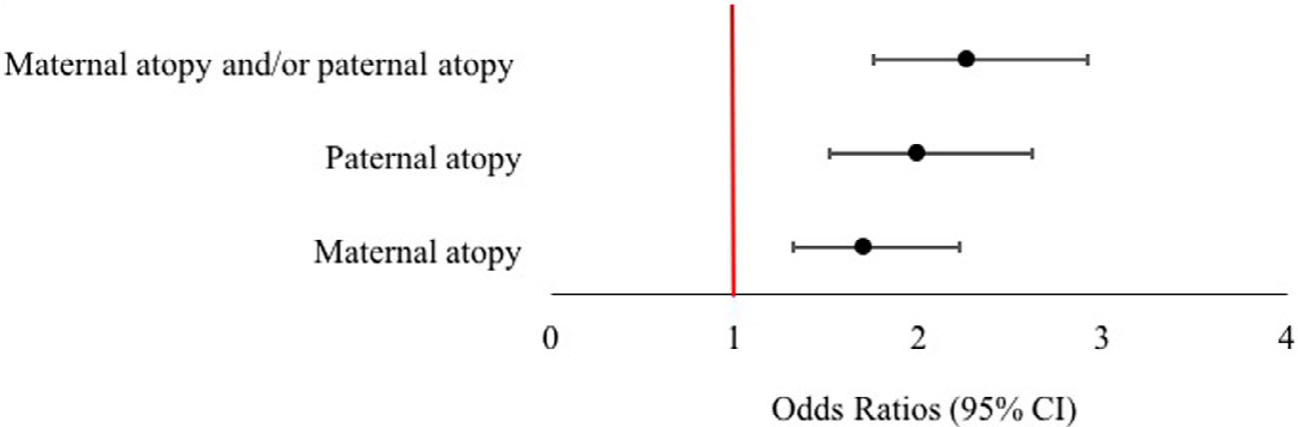
Plot showing odd ratios for development of atopic dermatitis in offspring in the first 2 years of life, according to maternal, paternal or biparental atopy (atopic dermatitis and/or asthma and/or rhinitis)

**FIGURE 3:**
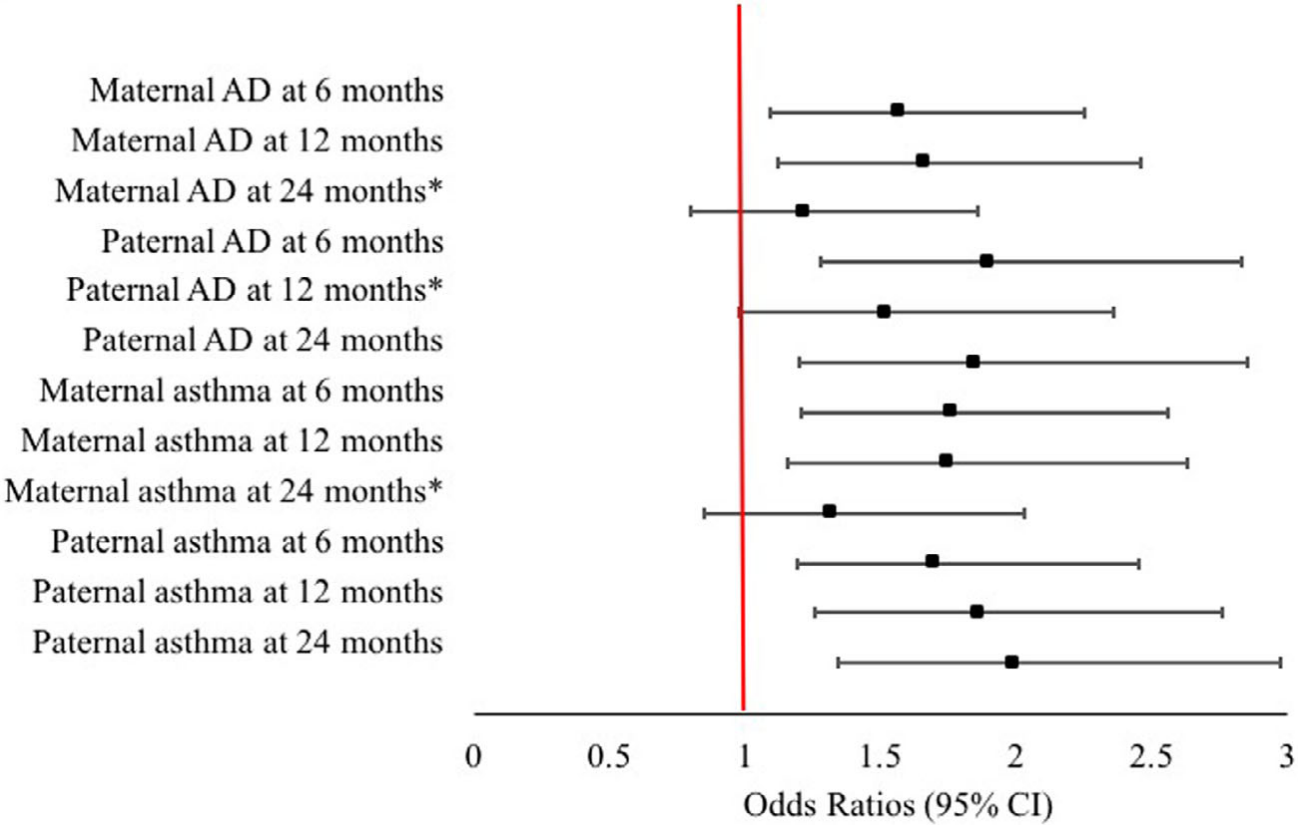
Plot showing odd ratios for development of atopic dermatitis in offspring at 6, 12, and 24 months, according to parental atopic dermatitis or asthma. *not statistically significant

The odds ratios and confidence intervals for development of AD in offspring at all‐time points with maternal and paternal AD and asthma are shown in Figures 2 and 3.

## 4 DISCUSSION

This secondary data analysis of a large unselected first‐born cohort showed an increased risk of AD in those who had a parental history of atopy, and specifically a maternal or paternal history of AD or asthma. These results were consistent even after accounting for confounding factors, such as birth weight and transepidermal water loss (TEWL) at 2 months. This study showed a similar or slightly higher impact of paternal atopy on AD development, compared to maternal atopy. Parental allergic rhinitis was not associated with AD in offspring in the first 2 years, except for maternal rhinitis at 24 months. The genetic predisposition to allergic rhinitis, given the key role of aeroallergen sensitization in its pathogenesis, may not be associated with early onset AD, but may have a greater impact in later onset or persistent AD.

Strengths of this study include the large sample size, the unselected nature of the subjects, the detailed assessments, and the extended follow up. AD was assessed by trained researchers using validated diagnostic criteria. Follow up to 24 months excluded transient eczematous eruptions that are common in the first year of life, and most patients who develop AD do so by 24 months. Retention between 6 months (n = 1836) and 24 months (*n* = 1617) was high at 88.1%, although complete data on AD status were missing on 16.2% (298/1836) of infants at 6 months and 20.7% (335/1617) of subjects at 24 months. Confounding factors were controlled for as far as possible. Limitations include the fact that this was a secondary data analysis. Parental AD, asthma, and rhinitis were self‐reported, which may reduce reliability and may contribute to the differences seen between the impact of maternal and paternal reported atopy on offspring. Data on siblings were not captured, as participants in the study were first‐born children. Filaggrin mutational analysis was not performed, which would have provided richer detail in the analysis of parental history and TEWL. Children who developed medical problems such as AD may have been more likely to stay in the study, and children without health problems may have been more likely to be lost to follow up, which may skew the prevalence data or other results.

Most previous studies have used parent‐reported data for diagnosis of atopy in both parents and children.^7^, ^8^, ^9^ Very few studies have used objective assessment of AD for diagnosis in offspring. The prevalence of AD in children has increased dramatically in recent years,^16^ and most studies reporting the impact of parental atopic history on AD are based on older data. Given the recent interest in early intervention to prevent AD and other allergic diseases,^17^ enhanced early identification of infants at risk of AD is increasingly important. This study provides a detailed analysis of the risk of AD associated with parental atopy at multiple specific time points in early life, which may help to risk stratify infants to optimize early interventions for prevention or early treatment of AD.

This study from a longitudinal birth cohort adds to the knowledge base relating to parental atopy and risk of AD, showing that both maternal and paternal histories of AD and asthma are associated with increased risk of AD in offspring in early life.

## AUTHOR CONTRIBUTIONS

Cathal O'Connor co‐designed the study, analyzed and interpreted the data, wrote the first draft, and reviewed the manuscript. Vicki Livingstone, Jonathan O'B. Hourihane, Alan D. Irvine, and Geraldine Boylan analyzed and interpreted the data and reviewed the manuscript. Deirdre Murray co‐designed the study, analyzed and interpreted the data, and reviewed the manuscript. All authors gave final approval of the version to be published.

## FUNDING INFORMATION

This publication has emanated from research supported in part by a research grant from Science Foundation Ireland (SFI) under Grant Number 12/RC/2272 and 15/SP/3091 and Johnson & Johnson. The Cork BASELINE Birth Cohort Study (ClinicalTrials.gov NCT01498965) is supported by the National Children's Research Centre, Dublin, Ireland and by the Food Standards Agency, United Kingdom (TO7060). Cathal O'Connor is funded by the Irish Clinical Academic Training (ICAT) program, supported by the Wellcome Trust and the Health Research Board (grant number 223047/Z/21/Z); the Health Service Executive National Doctors Training and Planning; and the Health and Social Care, Research and Development Division, Northern Ireland.

## CONFLICT OF INTEREST

JO'BH receives research funding related to this field from the City of Dublin Skin and Cancer Hospital Charity, and to unrelated research projects from Clemens von Pirquet Foundation and Temple St Hospital Research Foundation and Johnson & Johnson, research funding and speaker fees and consultancy fees from Aimmune Therepeutics, research funding and speaker fees from DBV Technologies.

## Data Availability

The data that support the findings of this study are available on request from the corresponding author. The data are not publicly available due to privacy or ethical restrictions.
